# Characterization and short-term culture of cells recovered from human conjunctival epithelium by minimally invasive means

**Published:** 2009-10-27

**Authors:** Hernán Martínez-Osorio, Margarita Calonge, Alfredo Corell, Roberto Reinoso, Antonio López, Itziar Fernández, Eloína Gutiérrez San José, Yolanda Diebold

**Affiliations:** 1 Ocular Surface Group, Institute for Applied Ophthalmobiology (IOBA), University of Valladolid, Edificio IOBA, Campus Miguel Delibes, Valladolid, Spain; 2 Networking Research Center on Bioengineering, Biomaterials and Nanomedicine, Valladolid, Spain; 3 Immunology Department, University of Valladolid, Ramón y Cajal 7, Valladolid, Spain

## Abstract

**Purpose:**

To characterize conjunctival cells obtained by brush cytology (BC) and establish short-term cultures.

**Methods:**

Human tarsal and bulbar conjunctival cells were obtained by BC and transported in 3 different media: serum-free medium (DK-SFM) with low [Ca^2+^], 10% fetal bovine serum (FBS) supplemented medium (FBSm10), and 20% FBS-supplemented medium (FBSm20). Recovered cells were counted and initial viability assessed. Flow cytometry established epithelial or immune lineage, viability, apoptosis, and cell cycle stage. To establish short-term cultures, tarsal conjunctival cells were seeded onto Permanox™ or denuded amniotic membrane (dAM) and cultured in the 3 media. Living adherent cells were assessed on Days 1, 2, and 5 by fluorescence microscopy.

**Results:**

Initial cell recovery was significantly lower with DK-SFM than in the other two culture media. Flow cytometry showed that 3.8±0.4% of recovered tarsal cells were CD45+ leukocytes and 67.9±1.6% were CK7+ secretory epithelial cells. S-phase cells composed 3.5±0.3% of the recovered tarsal cells and 2.1±0.2% of the bulbar cells (p=0.0006). The percentage of viable, apoptotic, and dead cells was similar for tarsal and bulbar cells. Two different cell populations were observed in both locations. About 24% consisted of smaller, less complex cells with high viability, and the remainder was composed of larger, more complex cells with poor viability. Significantly more living cells were supported by FBSm10 on the dAM substratum (p*=*0.011) than by the other media on either dAM or Permanox.

**Conclusions:**

Conjunctival BC recovers proliferating cells that can be maintained on dAM in FBSm10 for up to 5 days.

## Introduction

Non-invasive methods to obtain cells for clinical and experimental cultures have been established for tracheobronchial epithelial cells [1,2], nasal epithelial cells [3], urothelial cells [4], umbilical endothelial cells [5], and squamous esophageal cells [6]. For ocular tissues, conjunctival impression cytology (IC) and epithelial cell culture are tools that allow laboratory investigation of the pathophysiological processes affecting the conjunctiva [7,8]. In contrast to biopsies [8], IC is a minimally invasive technique that collects the superficial 2 to 6 conjunctival cell layers for use in a wide range of techniques [7,9]. Brush cytology (BC) has been used for the same purpose and can deliver larger cell numbers than IC directly into suspension [10-14].

Compared to IC, BC recovers a more viable cell population (Diebold Y, et al. IOVS 2007;48:ARVO E-Abstract 5314). However, no reports on the establishment of epithelial cell cultures derived from BC samples have been published to date, but the in vitro maintenance of BC-collected cells for 24 h [15] or 72 h to perform biochemical analysis [16]. The aim of the present study was to determine the best transport medium for BC-recovered human conjunctival cells and characterize freshly isolated cells with respect to viability, lineage, and cell cycle. Further, we established short-term cultures to determine which medium and substratum best supports the recovered epithelial cells.

## Methods

### Subjects

This study was approved by the Institutional Review Board of the University of Valladolid and followed the tenets of the Declaration of Helsinki. Informed consent was obtained from every patient. We performed BC on the conjunctiva of one eye from 93 healthy donors (n=78 tarsal and 15 bulbar; mean age ± standard deviation: 63 ± 2 years). Donors were cataract surgery patients who were otherwise healthy and had no systemic or previous ocular disease other than cataract.

### Brush cytology

BC samples were always taken by the same person (HMO) after two drops of topical anesthesia (lidocaine 0.2%) were applied to the eye. To maximize the recovery of proliferating cells, IC was first performed to remove the most superficial and postmitotic cells. Briefly, one quarter of a polyethersulfone filter (pore size 0.2 μm; diameter 13 mm; Gelman Laboratory, Supor^®^ 200, Ann Arbor, MI) was applied to the superior tarsal and bulbar conjunctivas. Staining of the filters by our modified PAS-Papanicolau technique [7] showed that up to two cell layers from the bulbar conjunctiva and one cell layer of the tarsal conjunctiva were removed (data not shown). Immediately afterwards, at the same spot, BC was performed with one rotation of the Cytobrush^®^-Plus GT (Medscand Medical AB, Molmö, Sweden). The epithelial cells were then detached from the brush by gentle rotation for 30 s in an Eppendorf tube containing 1.4 ml of one of the three transport and culture media (described below). BC was then performed three more times at the same location.

For microscopic analysis, slides of recovered cells (n=5 tarsal and 5 bulbar) were prepared in duplicate by cytocentrifugation with a Shandon Cytospin (Southern Products, Astmoor, Runcorn, Cheshire, UK). Cells suspended in 400 µl of culture medium were centrifuged at 800 rpm for 5 min through a hole in a filter paper strip mounted on a glass slide. The filter paper absorbed the medium, and the cells adhered to a 6 mm diameter circular area of the slide. For each pair of slides, one was stained with hematoxylin/eosin and the other was immunostained anti-cytokeratin 7 (CK7) antibody as previously described [17].

### Transport and culture media

All chemicals were purchased from Sigma-Aldrich (St Louis, MO, USA) unless otherwise indicated. Plastic culture dishes were from Nunc (Roskilde, Denmark), and basic cell culture media (Defined keratinocyte serum-free medium (DK-SFM) and the 1:1 mixture of Dulbecco’s modified essential medium and Ham F12 medium (DMEM/F12) and supplements were from Gibco-Invitrogen (Inchinnan, UK). Three different media with different total Ca^2+^ concentration (Table 1) were specifically designed to transport cells from surgery and to establish the cultures: (1) DK-SFM was a serum-free medium, (2) DMEM/F-12 was supplemented with 10% fetal bovine serum (FBS) (FBSm10), and (3) DMEM/F-12 was supplemented with 20% FBS (FBSm20). Cells detached from the brush were dispersed by gentle pipetting, and 200 µl aliquots were used to determine cell number and viability by Trypan blue dye exclusion in a Neubauer chamber [8].

**Table 1:** Culture media.

**Media components**	**DK-SFM**	**FBSm10**	**FBSm20**
Basic medium	DK-SFM	DMEM/F12	DMEM/F12
Ca^2+^ Concentration [mM]	0.09	1.16	1.2
Supplements			
Bovine pancreas insulin [mg/ml]	+	1	5
Epidermal growth factor [ng/ml]	+	2	10
Cholera toxin [mg/ml]	-	0.1	0.1
Hydrocortisone [mg/ml]	-	0.5	0.5
Fetal bovine serum [%]	-	10	20
Penicillin [U/ml], streptomycin [mg/ml], amphotericin B [mg/ml]	-	50, 50, 2.5	50, 50, 2.5
bFGF	+		

+ indicates presence of the agent but the concentration was not provided by the vendor; bFGF: basic fibroblast growth factor.

### Flow cytometry

Cells recovered by BC from the superior tarsal and bulbar conjunctivas (n=10 each) were analyzed by flow cytometry (FCm) to establish lineage and vital state. Cell counts and viability in FBSm10 were determined in 200 µl aliquots. The remaining cells were processed for three different analyses: cell lineage, apoptosis, and cell cycle. For these assays, 11±0.1×10^3^ events (mean±SEM, range: 5.0×10^3^–20.0×10^3^) were acquired and the whole cell samples were analyzed. All cytometry assays were performed with the same threshold for size (channel 100 of Forward Scatter [FS]). Cell fluorescence was measured using a Cytomics FC 500 Cytometer (Beckman-Coulter, Fullerton, CA). An argon-ion laser (488 nm) and a HeNe laser (633 nm) were used to excite phycoerythrin-cyanin 7 (PC7, red), propidium iodide (PI, orange), and fluorescein isothiocyanate (FITC, green) fluorochromes. Mean Fluorescence Intensities (MFI) were quantified and expressed as arbitrary units. The MFI value is directly related to the density of a given protein per cell [18]. IOBA-NHC cells [17] (passage 78–85) were used to standardize the protocols for each cell lineage analysis, apoptosis assay, and cell cycle analysis (data not shown). Controls included cross-reactivity of the fluorescence signals of each channel, as well as matched unspecific monoclonal antibodies (mouse) used as isotype negative controls (Table 2). All samples were analyzed in a masked fashion by the same examiner (EGSJ).

**Table 2:** Reagents for flow cytometry.

**Reagent**	**Specificity**	Channel*
CK7-FITC mAb (clone LP5K)	Secretory epithelium	FL-1
CD45-PC7 mAb (clone J33)	Peripheral blood leukocytes, APC and PMN cells	FL-5
AnnexinV-FITC	Phosphatidylserine, which is translocated from the inner to the outer leaflet of the cell membrane in early apoptosis process	FL-1
PI in apoptosis assay	DNA of cells with permeable membranes	FL-3
PI in cell cycle assay	DNA of cells in which membranes were permeabilized in the protocol	FL-3
IgG2b-FITC mAb	Isotype negative control	FL-1
IgG1- PC7 mAb	Isotype negative control	FL-5

CK7: Cytokeratin 7; CD45: Leukocyte common antigen; mAb: Monoclonal antibody; FITC: Fluorescein isothiocyanate; PC7: Phycoerythrin-cyanin 7; APC: Antigen-presenting cell; PMN: Polymorphonuclear; PI: Propidium iodide. The asterisk indicates that each channel reads a specific fluorochrome: FL-1, FITC; FL-3, PI; FL-5, PC7.

#### Cell lineage analysis

To determine the nature of the recovered cell types, 400 µl of cell suspension was analyzed by direct immunofluorescence using the following monoclonal antibodies (Table 2): PC7-labeled anti-CD45 (CD45-PC7; Beckman Coulter, Marseille, France) as a pan leukocyte marker [13] and FITC-labeled anti-cytokeratin-7 (CK7-FITC; Chemicon International, Temecula, CA) as a marker of secretory epithelium [19]. Cell suspensions were incubated first in the dark with CD45-PC7 antibody at room temperature (RT) for 20 min. They were then incubated in the dark with 1 ml of FACS^TM^ Lysing solution (BD Biosciences, San Jose, CA) at RT for 10 min to fix the cells. After washing with 2 ml of cell wash solution (BD Biosciences), the epithelial cells were then centrifuged at 500× g for 5 min. After removal of the supernatant, the pelleted cells were resuspended with 500 μl of permeabilization solution (1:10, FACS^TM^ Permeabilizing Solution 2; BD biosciences) for 10 min in the dark at RT. The cells were washed with cold phosphate buffered saline (PBS, 2 ml) and centrifuged again at 500× g for 5 min. The supernatant was removed and cells were incubated with CK7-FITC antibody (10 μl) at RT in the dark for 30 min. Finally, cells were washed with 2 ml of PBS, and centrifuged at 500× g for 5 min. The supernatant was removed, and the cells were fixed in cold PBS containing 1% p-formaldehyde for flow cytometry analysis.

#### Viability, apoptosis and cell death detection

To determine viability, apoptosis, and cell death, BC samples (300 µl) were processed using an annexin V-FITC and propidium iodide (PI) commercial kit (Beckman Coulter, Marseille, France). Following the manufacturer’s protocols, cell suspensions were washed with cold PBS (2 ml) and centrifuged at 500× g for 5 min. The supernatant was removed and the cells were resuspended with 100 μl of annexin binding buffer at 4 °C. Cells were then incubated at 4 °C in the dark with 1 μl of annexin V-FITC (25 μg/ml) and 5 μl of PI (250 μg/ml) for 10 min. Finally, 400 μl of binding buffer at 4 °C was added, gently agitated, and flow cytometric analysis performed. Annexin V is an anticoagulant that binds to negatively charged phosphatidylserine. Phosphatidylserine is an internal plasma membrane phospholipid that in the early apoptotic cascade is exposed on the outer layer of the plasma membrane, before the cell becomes permeable to PI. Therefore, vital and early apoptotic cells with intact cell membranes showed no fluorescent signal for PI. Early apoptotic cells stained only with annexin V, and double staining with annexin V and propidium iodide detected late apoptosis. Necrotic cells stained only with propidium iodide, and double negative cells were viable cells.

#### Cell cycle analysis

To study cell cycle, the remaining 300 µl of the BC samples was used to determine DNA content. After the cell suspension was centrifuged at 500× g for 5 min, the supernatant was removed and cells were permeabilized in 50 μl of DNA-Prep LPR (Coulter® DNA Prep^TM^ Reagents Kit; Beckman Coulter) at RT. DNA-Prep Stain^TM^ containing PI and RNA-ase III-A (1 ml) was added, gently agitated, and incubated for 30 min at RT in the dark, according to the manufacturer’s instructions. The preparations were stored in the dark before the analysis.

### Establishment of short-term cell cultures

Based on our results from the FCm study, tarsal conjunctiva was selected as the optimal site to obtain cells for culture. Approximately 10 min after the last brushing, cells were resuspended in 1 ml and then seeded onto either Permanox™ (Nunc), a standard plastic surface for cell culture (area: 4.2 cm^2^), or denuded amniotic membrane (dAM, area: 3 cm^2^) in a two-well plate. The amniotic membrane (gently provided by Banco de Ojos, Clínica San Francisco, León, Spain) was previously denuded by enzymatic digestion for 30 min with 1.2 U/ml Dispase (Sigma-Aldrich), followed by gentle scraping to remove any remaining epithelial cells [20]. The culture medium was changed every 2–3 days. Cells were incubated at 37 °C in a 5% CO_2_ atmosphere and were observed daily by phase contrast microscopy.

Living and dead cells were assessed on Days 1, 2, and 5 using the Calcein/EthDIII kit (Biotium Inc., Hayward, CA). Briefly, epithelial cells were washed twice with PBS, incubated with working solution composed of 2 mM Calcein AM and 4 mM EthD-III in PBS for 40 min at RT in the dark. The incubation solution was aspirated, and the cells were washed twice with PBS and mounted on slides. Cells in 5 fields (10×) were counted using a Leica DMI6000B digital fluorescence inverted microscope (Leica Microsystems, Mannheim, Germany). Three experiments were run with the 3 different culture media, 2 different substrata, and 3 time periods, making a total of 54 experiments. Cell counts were done twice and then averaged. Living cells grown on dAM in FBSm10 were identified on Day 5 by immunofluorescence using anti-CK7 and anti-CD45 antibodies (n=9).

### Statistical analysis

Statistical analysis was done by a Biostatistician (coauthor I.F.) using the SAS Proc Mixed procedure (SAS System for Windows Release 8.01; SAS Institute Inc., Cary, NC). The percentages of events obtained by FCm analysis were expressed as means±SEM. Statistical significance for flow cytometric data was determined by Student’s *t-*test for paired values. A repeated measures analysis of variance (ANOVA) followed by Tukey’s post-hoc test was used to analyze multiple pairwise comparisons between-subject main effects, and Bonferroni’s multiple comparison correction was used for within-subject and interaction effects.

## Results

### Initial cell recovery

Based upon Trypan blue exclusion by cells counted in the Neubauer chamber, the average of number of tarsal conjunctival cells recovered by BC in the DK-SFM transport medium, 12.1±0.3×10^4^, was significantly lower than in either FBSm10 or FBSm20 (14.5±0.6×10^4^ and 14.3±0.6×10^4^, respectively, p=0.036); however the initial viability of the cells recovered in the DK-SFM, 19.6 ± 0.6%, was not different from the other 2 media (FBSm10: 20.9±0.8% and FBSm20: 19.6±0.7%). Recovery of tarsal conjunctival cells in FBSm10, 13.0±0.5×10^4^, was not significantly different from bulbar conjunctival cell recovery, 16.0±1.6×10^4^. There were no significant differences in the viability of cells collected from these two areas. Hematoxylin/eosin staining of recovered cell slides prepared by cytocentrifugation showed normal cell morphology, and no differences between tarsal and bulbar cells was observed (data not shown). All recovered tarsal and bulbar epithelial cells were positive for CK7 as determined by immunofluorescence of the cytocentrifugation specimens. We did not attempt to identify leukocytes or others cells in the cytocentrifugation studies.

### Flow cytometry analyses

**Figure 1:**
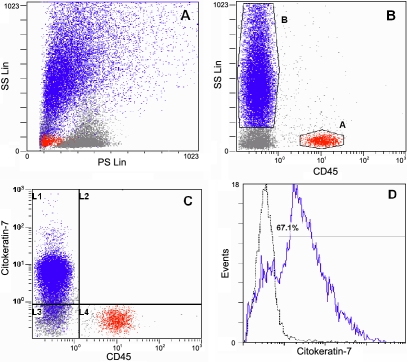
Cell lineage analysis determined by flow cytometry. **A**: Forward scatter (FS) versus side scatter (SS) plot showing conjunctival tarsal cells recovered by brush cytology (BC) in 10% fetal bovine serum (FBS) supplemented medium (FBSm10). **B**: CD45 versus SS dot plot showing a leukocyte population (gate A, red), epithelial cells (gate B, blue) and cellular debris (gray dots). **C**: CD45 versus CK7 dot plot showing a CD45+CK7- leukocyte population (region L4), CK7+CD45- epithelial cells (region L1) and CK7-CD45- cellular debris (region L3). **D**: Analysis of CK7 expression (blue line) over total BC-recovered cells; 67.1% of the cells were positive (isotype-matched negative control in dotted line).

Only cells transported to the laboratory in FBS10m were used for flow cytometry analysis. We selected this medium because of the high cell recovery determined in the Neubauer chamber and our previous success in growing IOBA-NHC cells with it [17]. Most recovered tarsal and bulbar conjunctival cells, 67.8±1.6% and 65.7±2.5%, respectively, (Figure 1), were positive for CK7, indicating that they consisted of secretory epithelial cells. While the percent composition of cells bearing CK7 was similar for the two conjunctivas, cells of bulbar epithelium had a greater density of staining as measured by the MFI, 7.5±0.8, than did cells of the tarsal epithelium, 4.6±0.4 (p=0.005, Table 3). The CD45 marker for intraepithelial leukocytes (Figure 1) was expressed in 3.8±0.4% of the tarsal cells and 2.8±0.4% of the bulbar cells (p>0.05, Table 3). Thus, most of the recovered cells were of epithelial origin in both locations.

**Table 3:** Cell lineage analysis.

**Origin**	**CK7**		**CD45**	
	**%**	**Density**	**%**	**Density**
Tarsal conjunctiva	67.9±1.6	4.6±0.4	3.8±0.4	1.1±0.0
Bulbar conjunctiva	65.7±2.5	7.5±0.8	2.8±0.4	1.3±0.2
p	0.48	0.005	0.07	0.14

The table summarizes the results of immunophenotyping of brush cytology-recovered cells from healthy human epithelium. CK7 and CD45 expressions on tarsal and bulbar conjunctival cells were measured as percentage of positive cells (%) and Mean Fluorescence Intensity (MFI, Density). Flow cytometric analysis showed that 3.8±0.4% and 2.8±0.4% of the tarsal and bulbar cells, respectively, were CD45+ intraepithelial leukocytes indicating that the recovered population was not purely epithelial (CK7+CD45-).

**Figure 2:**
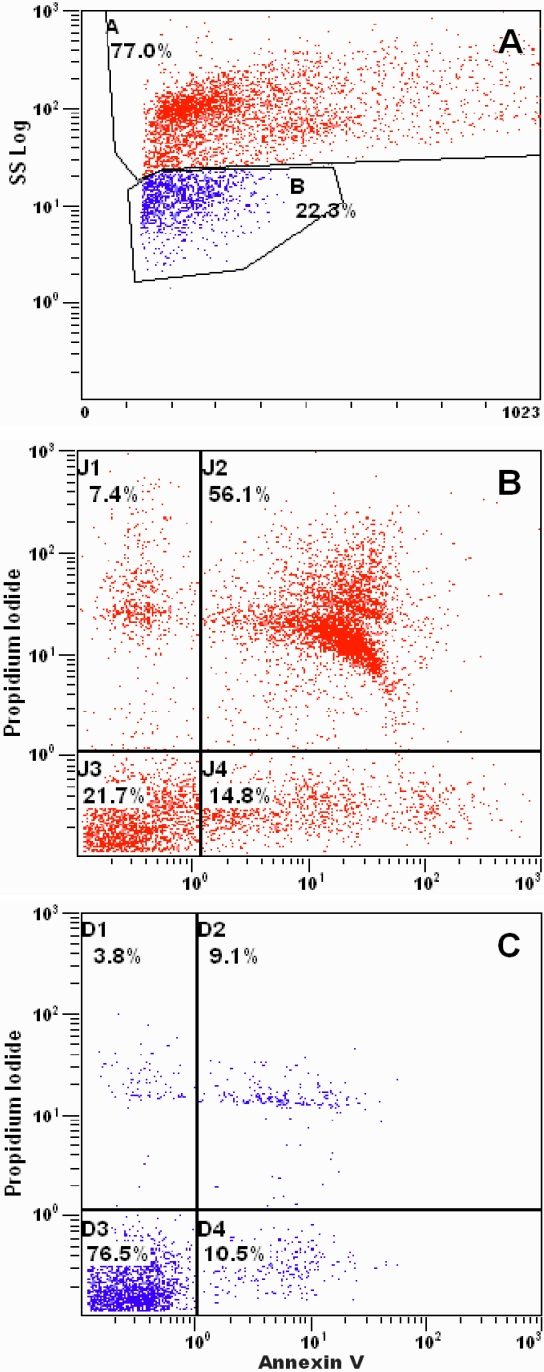
Tarsal conjunctival cell viability, apoptosis, and cell death determined by flow cytometry. **A**: The morphological characteristics of recovered tarsal cells defined two populations: larger, more complex cells (red) and smaller, less complex cells (blue). Side scatter/forward scatter was used to define the size/complexity of the cell pool. **B**: In this representative experiment, early apoptotic cells (J4) and late apoptotic cells (J2) composed 70.9% of the population of larger, more complex cells stained with propidium iodide (PI) and annexin V, consistent with high level of apoptosis. **C**: In a population of smaller, less complex cells, 76.5% (D3) had negative staining with PI and annexin V, indicating high viability.

In the apoptosis assay, cells were analyzed combining the standard FCm morphological parameter named complexity (cytoplasmic granularity) with cell size and viability (Figure 2A). Based on these criteria, the majority of tarsal conjunctival cells, 75.5±1.8%, were larger, more complex, with only 20.8±2.4% viability (Figure 2B, Table 4). This was consistent with the viability estimated by Trypan blue exclusion. Approximately 23.6±1.8% of the cells were smaller, less complex and had a viability of 82.0±2.4% (Figure 2C, Table 4). For bulbar cells, 77.4±2.6% were larger, more complex, and had a viability of only 21.8±3.8% (Table 4). This was also consistent with the viability estimated by Trypan blue exclusion. Approximately, 21.9±2.7% of the bulbar conjunctival cells were smaller, less complex and had a viability of 73.8±6.4% (Table 4). The more complex cells, most of which are apoptotic (Table 4), represent epithelial cells obtained after brushing. The less complex cell proportion largely exceeds the obtained percentage of CD45+ cells in both anatomic locations (tarsal and bulbar); thus, they may account for a mixture of both, epithelial and CD45+ cells.

**Table 4:** Viability, apoptosis, and cell death analysis.

**Origin**	**More Complex**				**Less Complex**			
	**% live**	**% early apoptosis**	**% late apoptosis**	**% dead**	**% live**	**% early apoptosis**	**% late apoptosis**	**% dead**
Tarsal conjunctiva	20.8±2.4	11.0 ±1.5	53.6±4.0	14.5±3.1	82.0±2.4	5.8±0.8	6.8±1.8	5.2±1.5
Bulbar conjunctiva	21.8±3.8	16.0 ±3.3	54.3±4.37	7.8±0.9	73.8±6.4	4.9±0.9	14.4±3.8	6.9±2.6
p	0.82	0.18	0.9	0.06	0.24	0.44	0.09	0.56

The table shows the percentages of live, apoptotic (early and late) and dead cells in two different cell populations (more and less complex) obtained from tarsal and bulbar human conjunctival epithelium. Most of the brush cytology-recovered cells (>75%) were larger, more complex and had poor viability (20.8±2.4% and 21.8±3.8% in tarsal and bulbar cells, respectively). The remaining cells (<25%) were smaller, less complex but with higher viability (82.0±2.4% and 73.8±6.4 in tarsal and bulbar cells, respectively).

The percentages of the more complex tarsal conjunctival epithelial cells in early apoptosis, late apoptosis, and dead were 11.1±1.4%, 53.5±4%, and 14.5±3.1%, respectively, which were not significantly different from the bulbar epithelial cells (Table 4). Less complex tarsal and bulbar cells had lower percentages of early apoptosis, late apoptosis, and dead cells than the more complex cells. Less complex tarsal cells were 5.8±0.8%, 6.8±1.8%, and 5.2±1.5%, respectively. Bulbar cells had similar percentages (p>0.05, Table 4).

**Figure 3:**
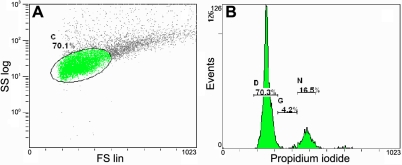
Tarsal cell cycle analysis by flow cytometry. **A**: When morphological cell parameters were analyzed by size and complexity, 70.1% of the cell population was homogeneous when observed in the flow cytometer gate C, which selects for cell cycle analysis of total cells. **B**: In flow cytometry gates D, G, and N, 70.3% were 2n cells (G_1_/G_0_), 4.2% were cells in S, and 16.5% were 4n cells (G_2_/M).

For statistical analysis of the cell cycle phases, a homogeneous cell population was selected (Figure 3A) based on the previous morphological analysis of total population. The sum of the percentages of cell phases of the homogeneous population was always greater than 80%. Cells in the S-phase of mitosis accounted for 3.5±0.3% of the tarsal epithelial cells, which was significantly greater than that of the bulbar epithelial cells, 2.0±0.2% (Figure 3B, Table 5). The percent of tarsal epithelial cells in G_1_/G_0_ and G_2_/M was 82.0±1.6% and 9.3±0.9%, respectively. Similar values were present for bulbar epithelial cells (Table 5).

**Table 5:** Cell cycle analysis.

**Origin**	**Cell cycle phase**		
	**G_1_/G_0_ (%)**	**S (%)**	**G_2_/M (%)**
Tarsal conjunctiva	82.0±1.6	3.5±0.3	9.3±0.9
Bulbar conjunctiva	84.4±0.9	2.1±0.2	8.5±0.5
p	0.21	0.0006	0.47

The table summarizes the cell cycle dynamic in the conjunctival epithelium from healthy donors. DNA content of brush cytology-recovered tarsal and bulbar cells was determined by analysis of the percentages of cells in the G_1_/G_0_, S and G_2_/M phases. The results showed that more than 80% of recovered cells were either quiescent or pre-cycling (2n) and less than 10% were in G_2_/M phases (4n). Flow cytometric analysis also showed that the percent of cells in S-phase was statistically higher in tarsal (3.5±0.3%) than bulbar (2.1±0.2%) epithelium.

### Short-term cell cultures

**Figure 4:**
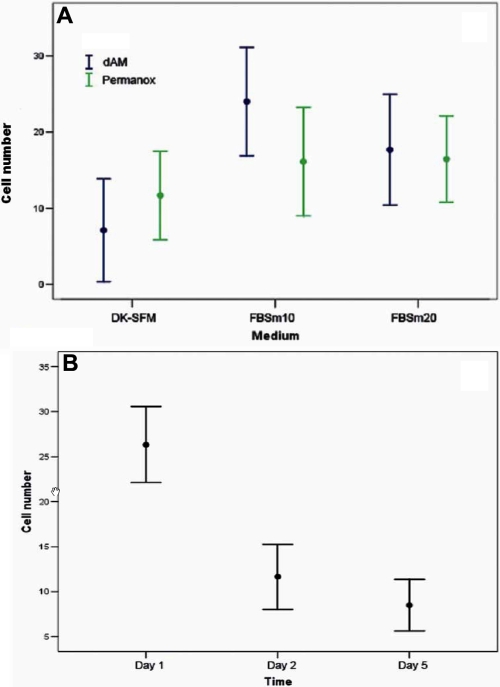
Effect of media, substrata, and time. **A**: Multiple comparisons analysis showed statistically more living tarsal conjunctival cells on Day 5 with 10% fetal bovine serum (FBS) supplemented medium (FBSm10) and denuded amniotic membrane (dAM) than the other media or substratum (p<0.011, n=9). **B**: Temporal evolution of cultures showed that the highest number of living tarsal conjunctival cells was on Day 1 and decreased (p<0.0001) on Days 2 and 5 (n=18), regardless of the substrate used. Values for **A** and **B** are means±95% confidence intervals.

**Figure 5:**
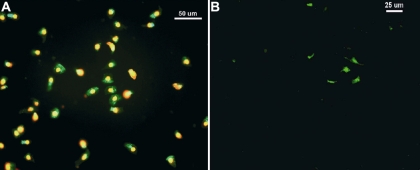
Effect of substratum on cell viability. On Day 5 of tarsal conjunctival cell culture in 10% fetal bovine serum (FBS) supplemented medium (FBSm10), the calcein dye was retained within living cells (green) while EthD-III was bound to nucleic acids of cells with damaged membranes (red). **A**: Numerous epithelial cells grown on Permanox™ showed red fluorescence typical of dead or dying cells. **B**: In contrast, cells grown on denuded amniotic membrane (dAM) showed less red fluorescence. Scale bar 50 μm (**A**) and 25 μm (**B**).

**Figure 6:**
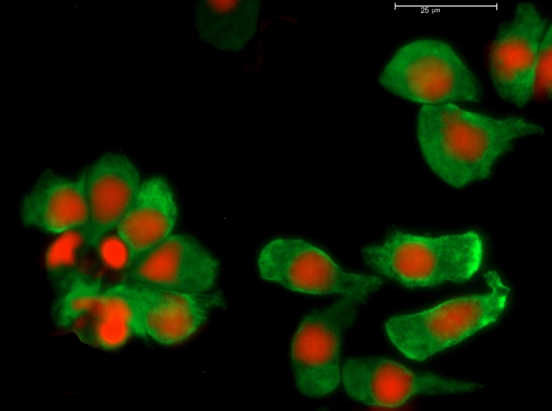
Cell lineage analysis by immunofluorescence. Double-labeling fluorescence with anti-CK7 (green) and anti-CD45 (blue) antibodies was used to identify CK7+ secretory epithelial cells and CD45+ leukocytes, respectively. Moreover, propidium iodide (PI) dye (red) was used to label nuclei. After 5 days of culture on denuded amniotic membrane (dAM) in 10% fetal bovine serum (FBS) supplemented medium (FBSm10), all conjunctival cells showed green fluorescence (CK7+ cells) with red nuclei (PI). In contrast, CD45+ cells were not observed in this culture (blue fluorescence). Scale bar 25 μm.

We selected superior tarsal conjunctiva as the source of cells to be maintained in culture because stem or transit amplifying epithelial cells are located here [21-24] and many diseases have clinical signs in this area [25,26]. Additionally, it is easy to take samples, and as we showed above, this area has the most S-phase cells. Living tarsal epithelial cells were maintained in culture for up to 5 days. The FBSm10 medium was significantly better than either DK-SFM or FBSm20 for supporting living cells in culture (Tukey p=0.007). Global analysis of the interaction effects showed the highest number of living cells occurred when FBSm10 and dAM were used together (Bonferroni p=0.011, Figure 4A, Table 6 and Table  7). The number of live cells decreased in culture over time (Tukey p<0.0001, Figure 4B) with the highest number present on Day 1 and decreasing on Days 2 and 5. For cells cultured on dAM in FBSm20, the survival ratio for living attached cells was 53.2% at Day 2 and 51.6% at Day 5. However compared to FBSm20, more living cells were attached at Day 5 with dAM and FBSm10 (Figure 5). At Day 5, all adhered cells cultured with FBSm10 on dAM had the morphological appearance of epithelial cells and were positive for CK7. CD45 positive cells were not observed (Figure 6).

**Table 6:** Number of tarsal conjunctival cells grown over dAM.

**Culture time**	**DK-SFM**		**FBSm10**		**FBSm20**	
	**Living cells**	**Dead cells**	**Living cells**	**Dead cells**	**Living cells**	**Dead cells**
Day 1	19.6±4.2	27.0±1.5	47.0±6.5	211.0±43.4	22.6±5.1	230.3±46.1
Day 2	1.0±1.0	30.0±6.5	22.6 ± 1.8	139.6±12.4	12.0±3.8	98.3±6.3
Day 5	0.6±0.3	28.0±4.9	16.0 ± 4.0	67.0±10.1	11.6±1.4	68.3±10.9

Abbreviations in the table are; DK-SFM: Defined keratinocyte serum-free medium; FBSm10: 10% fetal bovine serum (FBS) supplemented medium; FBSm20: 20% fetal bovine serum (FBS) supplemented medium. The table shows the individual and combined effects of medium (DK-SFM, FBS10m and FBS20m) and culture time (1, 2 and 5 days) on the viability of tarsal conjunctival cells cultured on denuded amniotic membrane (dAM) substratum. The results demonstrated that FBSm10 medium was significantly better than either DK-SFM or FBSm20 for supporting living cells in culture and that brush cytology-recovered cells from human conjunctival epithelium can be maintained on dAM for up to 5 days.

**Table 7:** Number of tarsal conjunctival cells grown over Permanox®.

**Culture time**	**DK-SFM**		**FBSm10**		**FBSm20**	
	**Living cells**	**Dead cells**	**Living cells**	**Dead cells**	**Living cells**	**Dead cells**
Day 1	21.0±2.9	31.6±6.2	28.0±5.0	233.3±4.6	26.6±3.7	195.3±34.9
Day 2	10.6±3.3	37.6±7.3	14.0±3.0	211.3±46.9	11.6±2.0	196.0±20.0
Day 5	3.3±1.7	15.3±2.3	7.6±0.9	30.0±2.3	11.6±0.3	45.3±11.4

Abbreviations in the table are; DK-SFM: Defined keratinocyte serum-free medium; FBSm10: 10% fetal bovine serum (FBS) supplemented medium; FBSm20: 20% fetal bovine serum (FBS) supplemented medium. The table shows the individual and combined effects of medium (DK-SFM, FBS10m and FBS20m) and culture time (1, 2, and 5 days) on the viability of tarsal conjunctival cells cultured on Permanox® substratum. The results demonstrated that FBSm10 and FBSm20 media were the most effective for supporting living cells in culture and that brush cytology-recovered cells from human conjunctival epithelium can be maintained on Permanox® for up to 5 days.

## Discussion

The development of non-invasive or minimally invasive techniques of collecting ocular surface cells for culture may provide improved methods for understanding the biologic and pathological features of many diseases. This kind of model blends the advantages of a non-invasive cell recovery with traditional cell culture techniques. Additionally, it will allow repeated studies in outpatients at different periods of the disease or the therapy. Previously, conjunctival cells obtained by BC were maintained in vitro for 3 days with the purpose of performing biochemical analysis [15,16]. In our study, we cultured epithelial conjunctival cells for 5 days by optimizing the culture medium and substratum. We also described new FCm data not previously available regarding normal conjunctival homeostasis.

We recovered 13×10^4^ conjunctival cells, which was higher than in other studies, even though we detached 1–2 cell layers by IC before performing the 4 brushings. Tsubota et al. [10-13] obtained 1×10^5^ cells after 20 brushes of the temporal bulbar conjunctiva. With an unspecified number of brush strokes, in some cases they obtained 1×10^6^ cells from pterygia and from normal conjunctiva without compromising the stroma or the basal membrane [27]. All bulbar conjunctival layers, including basal cells, can be removed after two consecutive ICs (Diebold Y, et al., IOVS 2007;48:ARVO E-Abstract 5314), and we assume that this can occur after 4 brushes.

The viability of our freshly isolated tarsal and bulbar epithelial cells, about 20%, was similar whether measured by Trypan blue or by flow cytometry. Similar viabilities were reported for bronchial epithelium [1] and umbilical endothelial cells collected by BC and deposited in FBS as the transport medium [5]. In our study, the initial cell recovery was significantly lower in the serum-free DK-SFM transportation medium than in the serum-containing media. The absence of serum may have accounted for an initial cell loss. This is consistent with the improved recovery of endothelial cells from artificial surfaces by the addition of 1% human albumin or 20% FBS to the isolation medium [5]. Serum, along with epidermal growth factor and glucocorticoids, reduces apoptosis in cultured epithelial cells and increases proliferation [28].

Tsubota et al. [12 reported that >99% of the cells recovered by BC from normal donors were epithelial] and other authors reported no lymphocyte morphological identification [10,13]. However, we found CD45 expression in 3.8% of the tarsal cells, most of which are probably lymphocytes due to their small complexity (Figure 1B). This proportion is similar to that obtained by IC [29].

We selected CK7 to stain conjunctival epithelium because it is expressed by epithelial cells in the conjunctiva [19]. All of the freshly isolated cells in the cytospin preparations expressed CK7. In contrast, only about two thirds of the tarsal and bulbar cells expressed CK7 by flow cytometry. This difference may be attributed to different sources of the antibody and different fixations used in each procedure. CK7 density was greater in the bulbar epithelial cells than in the tarsal ones (almost double), indicating a significant difference in their functional status, which may mean different i) cell activation, or ii) differentiation states. The population of epithelial cells that were recovered by BC consisted of a mixture of more differentiated cells, probably located near the apical surface of the epithelium, and less differentiated cells, probably located in the more basal regions of the epithelium. Tsubota et al. [11] also recovered basal conjunctival cells by BC.

The balance between proliferation and cell death determines the cellular homeostasis of every tissue. We studied both processes by flow cytometry and found differences from previous reports. Some studies reported that normal ocular surface epithelium has more cells in G_1_, followed by S, and lastly by G_2_/M phase [30]. However, we found more cells in G_2_/M than S, similar to findings of normal donors in Singapore described by Karukonda et al. [31]. The proliferative index of the freshly isolated tarsal epithelial cells was significantly higher than in bulbar samples. Both of these were lower than those obtained by ocular surface biopsies [30,31]. Normal conjunctival and pterygium epithelium were reported to have similar proliferation indexes [32], but all of these studies used different methods to obtain cells in suspension.

We found that 82.0% of tarsal and 84.4% of bulbar epithelial cells were in G_1_/G_0_. A previous report also established similar percentages in conjunctival biopsies of normal donors and pterygium patients [31]. These findings mean that close to 10% of the recovered cells were dividing, while more than 80% were either quiescent (G_0_) or pre-cycling (G_1_). Because there are proliferating cells in unwounded normal conjunctiva, it is likely that we obtained basal cells by BC, as others authors have done [11]. Some of these may have been conjunctival stem cells that have been described in different locations such as the muco-cutaneous junction [21], distal duct of meibomian glands [22], tarsal conjunctiva [23], fornix [24], and bulbar conjunctiva [24].

Another essential process in the maintenance of the ocular surface epithelium is apoptosis. We established that the percentages of early apoptotic and dead cells were similar. The percentage of dead cells was probably lower than expected because we detached and discarded the most superficial squamous layer by IC. In normal cells recovered by IC, the percentage of early apoptotic cells is around 30% [33], higher than we obtained. The brush procedure itself could explain the high percentage of late apoptotic cells, around 53%, in the more complex cells. A portion of these cells could have been in early apoptosis but the plasma membranes were possibly damaged by the brushing.

The two different cell populations, “more complex” and “less complex,” based upon cytoplasmic granularity and viability within the epithelium have not been reported previously. There are only two reports of apoptosis in samples obtained by non-invasive means [33,34], and neither of them have flow cytometry results similar to ours. Our hypothesis is that the less complex, more viable cells come from deeper levels of the epithelium, and probably are less differentiated. However, more work has to be done to establish this with certainty.

Culture conditions influence cell phenotype and maintenance of specific cell populations [2,20]. The FBSm10 medium was specifically designed to sustain the IOBA-NHC conjunctival epithelial cell line [17]. The FBSm20 medium has higher concentrations of insulin, EGF, and FBS, all of which enhance epithelial proliferation better than FBSm10. Boyce and Ham reported that primary cultures of epithelial cells increased proliferation in media with low Ca^2+^ concentrations, 0.05–0.3 mM, whereas higher Ca^2+^ concentrations, 0.9–1.8 mM, induced initial adhesion to the substratum and further stratification [35]. DK-SFM medium, with only 0.09 mM Ca^2+^, reduces fibroblast or melanocyte contamination and, according to the manufacturer, is designed for primary culture of skin, cervical, and oral epithelia. We have previously shown that DK-SFM culture medium delays proliferation of the IOBA-NHC epithelial cell line, even when dAM is used as the substratum (Martínez-Osorio H, et al. IOVS 2006;47:ARVO E-Abstract 4933). In the same way, DK-SFM medium induced the lowest level of conjunctival cell adhesion and proliferation when compared to the other two culture media. Although FBSm10 and FBSm20 were equally suitable to transport cells, FBSm10 was the better of the two in supporting adhesion of living cells and maintaining them for 5 days in culture.

The use of substrata to cultivate cells obtained by non-invasive methods is usually required [1-6]. For conjunctival epithelial cells, cultures over basement membrane and over amniotic membrane stroma have been widely described [20]. The basement membrane of the amniotic membrane resembles that of the cornea [36], conjunctiva [37], and limbal epithelium [38], and it maintains the normal phenotype of human ocular surface cells in culture [39]. For this reason, we used dAM as a substratum to culture epithelial cells. As a control we used Permanox™, a special solvent resistant resin that enhances cellular adherence and has very low autofluorescense. With dAM, the FBSm10 favored cell adhesion and maintenance of living conjunctival epithelial cells. At Day 5 of culture, the highest number of cells remained, which was superior to cultures plated on Permanox™ and with the other two media. Also, after 5 days in culture, those living cells maintained conjunctival epithelium markers (CK7 immunopositivity).

Adhesion depends not only on FBS percentage in medium but also, on the kind of substrate assayed and the amount present of the remaining supplements in the medium. Taking into account those parameters altogether, and in our culture conditions, FBS10m resulted superior to sustain initial viability of cells-to-be-plated and short-term culture maintenance. Regarding DK-SFM medium, most reports using this medium also used standard plastic surfaces to culture cells. Although our results using Permanox™ plus DK-SFM medium were slightly better than those of amniotic membrane plus DK-SFM medium, in terms of cell survival and growth, differences between them were not statistically significant. Besides, this medium was worse than the other two, regardless of the substrate. We do not have a hypothesis right now to explain why cells grown on Permanox and maintained in DK-SFM are slightly more viable than those grown on amniotic membrane. The exact concentration of each component of this medium is not provided by the manufacturer; this fact is a handicap when trying to explore different relationships with substrates. On the other hand, Permanox™ is reported to promote cell attachment through non-specific electrostatic interactions, while amniotic membrane may act through bioactive surface interactions in which serum components might interact. As DK-SFM medium has no serum, the combination Permanox™ plus DK-SFM may be advantageous for initial cell attachment.

Currently there is no ideal in vitro model of conjunctival epithelium for studying pathophysiological processes. Even though our results are encouraging, there are some aspects that need improvement. The recovery of epithelial cells by BC could be too low to establish long-term cell cultures, and brushing itself can damage the cell membrane, reducing the viability. Also, the recovered population is not purely epithelial. As we demonstrated, there are more CD45+ cells than previously reported. If a true differentiated monolayer of the conjunctival epithelium could be maintained for longer than 5 days, it would allow the performance of additional studies. For example, the epithelium could be co-cultured with other cell types such as dendritic cells, endothelial cells, mast cells, T cells or fibroblasts that participate in the maintenance and homeostasis of the ocular surface epithelium.

In conclusion, BC is a suitable technique to obtain a sufficient quantity of viable cells to establish initial short-term cell cultures. Whether these cells can be made to survive and proliferate beyond 5 days in culture has yet to be determined.
